# Right time, right place: improving access to health service through effective retention and distribution of health workers

**DOI:** 10.1186/1478-4491-11-60

**Published:** 2013-11-25

**Authors:** Ian Crettenden, Mario Dal Poz, James Buchan

**Affiliations:** 1 Health Workforce Australia, Adelaide, Australia; 2 Human Resources for Health

## Abstract

This editorial introduces the 'Right time, Right place: improving access to health service through effective retention and distribution of health workers’ thematic series. This series draws from studies in a range of countries and provides new insights into what can be done to improve access to health through more effective human resources policies, planning and management. The primary focus is on health workforce distribution and retention.

## Editorial

Several of the main human resources for health (HRH) issues in Australia will be familiar to readers in other countries: addressing health workforce geographic distribution, retention and productivity. This thematic series examines aspects of these common HRH challenges by drawing on studies from a range of countries. The series will provide new insights into what can be done to improve access to health through more effective human resources policies, planning and management, with a specific focus on health workforce distribution and retention. The papers in the series will be published over the next few months in the journal, and are timed both to coincide with the Third Global Forum on Human Resources for Health, in Recife, Brazil, from 10 to 13 November 2013, and the Health Workforce Australia (HWA) annual conference in Adelaide, Australia, from 18 to 20 November 2013. Health Workforce Australia (HWA) is pleased to support this new article collection in Human Resources for Health.

Health systems around the world are looking to improve access to health services and health system effectiveness. Two key interlocking components of a sustainable health workforce solution are to keep scarce skills in the system by effective retention strategies, and to enable them to be deployed where they can best make a positive difference to population health. The dynamic nature of health care labor markets mean that retention and distribution policies must be aligned with changing health workforce profiles, as well as with health system priorities, and be kept under periodic review to assess their impact.

Retention and geographic distribution are both high on the agenda in most countries, including Australia, with an increasing recognition that they are critical aspects of achieving effective health service delivery, particularly in rural and remote areas [1]. It also recognized that health workforce stability can contribute to improved outcomes. This linkage is even more pronounced in the context of the current debate about how all countries can achieve universal health coverage (UHC), with key UHC dimensions of access, availability and acceptability being predicated on having the health workforce, with the right skills – 'at the right time, in the right place’.

Many countries have tried a variety of incentives or regulations to influence or direct the choice of practice location of physicians. Such policies have often been categorized by reviewers under three or four main areas of intervention: education; financial incentive; regulation; or service delivery reorganization [2,3]. These same reviews also emphasize the need to look at 'bundling’ or sequencing of different policy interventions if sustained improvements are to be achieved [4]. They also highlight a general lack of follow-up assessment or evaluation of their implementation, which limits the ability of policy makers to use available resources most effectively in improving retention and distribution.

It is against this backdrop of high and growing policy emphasis on improving health workforce retention and distribution, coupled with low and fragmented evidence on policy intervention, that this article collection is being published in '*Human Resources for Health*’. The aim is to report on new analysis, strategic intelligence, and evidence that is pointing to improvements in retention and distribution of health workers around the world.

HWA is a federal agency that coordinates a national approach to the planning, training and deployment of the Australian health workforce. Addressing broader health workforce challenges, HWA is building the capacity, boosting the productivity and improving the distribution of the health workforce to make it more affordable and sustainable. HWA recognizes the critical importance of effective retention and distribution policies in meeting these challenges and has a current focus on the medical and nursing workforce. From a distribution perspective, HWA’s work is guided by an overarching rural and remote workforce innovation and reform strategy which is a blueprint to tackling key issues in the delivery of rural and remote health care [1].

With a key challenge around the maldistribution of the workforce, HWA is assessing the effectiveness of current measures to manage and achieve improvements in the distribution of medical practitioners in Australia. This work links to the current Organization for Economic Co-operation and Development (OECD) project which is examining the policy environment of geographic distribution of doctors in OECD countries.

HWA also has a sharp focus on the nursing workforce following the release of its national projections which identified retention and productivity of nurses as significant factors in reducing the projected shortfall of up to 109,000 nurses by 2025 [5]. Identifying the priority issues to further improve nurse retention and productivity and identifying local programs and initiatives across Australia which are proving to be effective is a key focus of this work.

This article collection from *Human Resources for Health* will provide new insights for practitioners, policy makers and analysts who have a responsibility or interest in what can be done to improve access to health through more effective human resources policies, planning and management.

## Competing interests

The authors declare no competing interests. The peer review process was independent of the sponsoring organization.
